# Patient size matters: Effect of tube current modulation on size‐specific dose estimates (SSDE) and image quality in low‐dose lung cancer screening CT

**DOI:** 10.1002/acm2.12857

**Published:** 2020-04-06

**Authors:** Izabella Barreto, Nupur Verma, Nathan Quails, Catherine Olguin, Nathalie Correa, Tan‐Lucien Mohammed

**Affiliations:** ^1^ Department of Radiology College of Medicine University of Florida Gainesville FL USA

**Keywords:** image quality, lung cancer screening CT, radiation dose, size‐specific dose estimates, tube current modulation

## Abstract

**Purpose:**

We compare the effect of tube current modulation (TCM) and fixed tube current (FTC) on size‐specific dose estimates (SSDE) and image quality in lung cancer screening with low‐dose CT (LDCT) for patients of all sizes.

**Methods:**

Initially, 107 lung screening examinations were performed using FTC, which satisfied the Centers for Medicare & Medicaid Services' volumetric CT dose index (CTDI_vol_) limit of 3.0 mGy for standard‐sized patients. Following protocol modification, 287 examinations were performed using TCM. Patient size and examination parameters were collected and water‐equivalent diameter (*D_w_*) and SSDE were determined for each patient. Regression models were used to correlate CTDI_vol_ and SSDE with *D_w_*. Objective and subjective image quality were measured in 20 patients who had consecutive annual screenings with both FTC and TCM.

**Results:**

CTDI_vol_ was 2.3 mGy for all FTC scans and increased exponentially with *D_w_* (range = 0.96–4.50 mGy, R^2^ = 0.73) for TCM scans. As patient *D_w_* increased, SSDE decreased for FTC examinations (R^2^ = 1) and increased for TCM examinations (R^2^ = 0.54). Image quality measurements were superior with FTC for smaller sized patients and with TCM for larger sized patients (R^2^ > 0.5, *P* < 0.005). Radiologist graded all images acceptable for diagnostic evaluation of lung cancer screening.

**Conclusion:**

Although FTC protocol offered a consistently low CTDI_vol_ for all patients, it yielded unnecessarily high SSDE for small patients and increased image noise for large patients. Lung cancer screening with LDCT using TCM produces radiation doses that are appropriately reduced for small patients and increased for large patients with diagnostic image quality for all patients.

**Abbreviations**
AAPM: American Association of Physicists in Medicine
ALARA: As Low As Reasonably Achievable
AEC: automatic exposure control
BMI: body mass index
CMS: Centers for Medicare and Medicaid Services
CNR: contrast to noise ratio
CTDI_vol_: volumetric CT dose index
CXR: chest radiography
DLP: dose length product
*D_w_*: water‐equivalent diameter
FTC: fixed tube current
LDCT: low dose CT
HU: Hounsfield Unit
IRB: Institutional Review Board
mA: milliamperes
mAs: milliamperes‐second
NLST: National Lung Screening Trial
R^2^: Pearson correlation coefficient
ROI: region of interest
SSDE: size‐specific dose estimates
TCM: tube current modulation
USPSTF: US Preventive Services Task Force
WL: window level
WW: window width

## 1 Introduction

Lung cancer is the leading cause of cancer‐related death in the United States, with an estimated 234,030 new lung cancer cases and 154,050 lung cancer deaths in 2018.^1^ This high mortality is due to the asymptomatic nature of lung cancer, where the majority of patients seek medical care after symptoms have developed, often when lung cancer has progressed to more advanced stages.^2^ Because lung cancer has a higher survival rate when detected at an early stage, screening in high‐risk individuals provides a preventative approach to reduce the number of lung cancer deaths.^3^ Launched in 2002, the National lung screening trial (NLST) enrolled 53,454 high‐risk smokers to undergo three annual lung cancer screening exams with either standard chest radiography (CXR) or low‐dose CT (LDCT).^4^ In 2011, the group published that 20% fewer lung cancer deaths were observed in participants screened with LDCT rather than CXR, due to the improved sensitivity of CT and its ability to resolve small nodules.^5^

In 2013, the US Preventive Services Task Force (USPSTF) recommended annual lung cancer screening with LDCT,^6^, ^7^ and soon after in 2015, the Centers for Medicare and Medicaid Services (CMS) approved reimbursement for annual lung cancer screening with LDCT for eligible patients.^8^ Eligible patients include asymptomatic adults between 55 and 77 yr of age who had a smoking history of at least 30 pack‐years, who are current smokers or have quit within the last 15 yr. To support hospitals implementing lung screening with LDCT, the American College of Radiology introduced its Designated Lung Cancer Screening Center program in 2014^9^ and the Lung Cancer Screening Registry in 2015.^10^ Furthermore, the American Association of Physicists in Medicine (AAPM) published recommended lung cancer screening protocols for a variety of CT scanner manufacturers and models to facilitate the provision of this helpful screening to the larger population.^11^

Following the recommendations from these groups, our hospital implemented a lung cancer screening program with LDCT in March 2015. However, the initial scan protocol utilized a single fixed tube current (FTC) value for all patients. Over a 1‐year period, scan techniques were not modified for different patient sizes, despite statements from the CMS, AAPM, and ACR specifying that radiation doses must be reduced for smaller sized patients and increased for larger sized patients examined for lung cancer screening with LDCT.^8^, ^11^, ^12^

Today's CT systems offer automatic exposure control (AEC) with tube current modulation (TCM) to reduce dose to the patient while maintaining image quality.^13^, ^14^ Tube current modulation is routinely used in thoracic and abdomen‐pelvic CT imaging, where the tube current is increased in higher attenuating regions such as the shoulders, and decreased in lower attenuating regions such as the lungs. TCM also accounts for different patient sizes by delivering sufficient tube current for proper x‐ray photon transmission through each patient.^15^, ^16^, ^17^

In order to modulate dose as a function of patient size, the original FTC scan protocol was modified to utilize AEC (CARE Dose 4D, Siemens Healthineers). This particular AEC system performs automatic TCM according to the patient's size and attenuation changes measured in the x‐ray localizer projection images, together with real‐time attenuation measurements measured during each tube rotation.^18^, ^19^ The adaptation of the tube current is based on a user‐defined image quality parameter called the Quality Reference mAs, expressed as the tube current–time product (milliamperes‐second, mAs) divided by the pitch and is selected according to the diagnostic requirements for the study.

Assessment of dose and image quality between both protocols for patients of all sizes is important to provide patient‐customized scanning that balances reducing radiation dose while providing acceptable image quality. This is especially important with higher prevalence of obesity in populations where screening is being implemented, and the repetitive exposure to radiation in a screening program. This study evaluates the effect of TCM on patient dose and image quality for 394 lung cancer screening examinations with LDCT, which has unique requirements of balancing detailed visibility of lung parenchyma while performing screening examinations with doses as low as possible.

We first compare Size‐Specific Dose Estimates (SSDE) and clinical image quality between patients scanned with FTC and TCM techniques. Second, considering the lack of a formal assessment of radiation dose and image quality for clinical lung cancer screening examinations, this study aims to validate that the recommended TCM‐based AAPM scan protocol for a Siemens Sensation 16‐slice scanner produces acceptable CT dose indices and diagnostic image quality for a variety of patient sizes.

## 2 Materials and Methods

Initially, a standard chest CT protocol was modified to create a low‐dose lung cancer screening CT protocol. The tube voltage was reduced from 120 kVp to 100 kVp, and the tube current was modified to utilize a single fixed tube current value of 150 milliamperes (mA) for all patients. In order to modulate dose as a function of patient size, the FTC protocol was modified to utilize 120 kVp and TCM with a Quality Reference mAs of 25, as recommended by the AAPM.^11^ Scan protocols utilized before and after the modification are described in Table 1.

**Table 1:** Scan techniques used before and after clinical protocol modification.

Protocol	FTC	TCM
Scan mode	Helical	Helical
Tube Voltage (kVp)	100	120
CARE Dose4D	Off	On
Tube Current	Fixed: 150 mA	Modulating: Quality ref mAs 25
Rotation time (s)	0.5	0.5
Detector configuration	16 × 1.5 mm	16 × 1.5 mm
Pitch	1.2	1.2
CTDI_vol_ (mGy)	2.5	Variable

Abbreviations: CTDI_vol_, volumetric CT dose index; FTC, fixed tube current; TCM, tube current modulation.

### 2.1 Retrospective data collection

After performing clinical examinations using the TCM scan technique for an 18‐month period, a retrospective study was approved by our Institutional Review Board (IRB) with a waiver of informed patient consent. Scan acquisition parameters were recorded for 394 examinations conducted on a Siemens Sensation 16‐slice scanner between March 1, 2015 and August 10, 2017, including kVp, rotation time, fixed mA or Quality Reference mAs, volumetric CT dose index (CTDI_vol_), and dose length product (DLP). Patient medical record numbers were also recorded in order to identify patients who received more than one annual screening examination. Furthermore, patient weight and height were recorded, and body mass index (BMI) was calculated by dividing weight (in kilograms) by height (in meters) squared.

### 2.2 Determination of size‐specific dose estimates (SSDE)

CTDI_vol_ is a dose index that provides information about the scanner output for a standard condition.^20^, ^21^, ^22^, ^23^ However, the dose received by a patient depends on both patient size and scanner output. AAPM Report 204 introduced a new metric, the SSDE, which can be used to estimate average patient dose based on the CTDI_vol_ and linear patient size measurements.^24^ AAPM Report 220 describes an improved method that estimates patient size based on patient attenuation by introducing the water‐equivalent diameter (*D_w_*).^25^ It is important to consider *D_w_*, rather than linear dimensional measurements in regions of the thorax, where attenuation is reduced.^25^

Size‐specific dose estimates was determined for each patient using [eq. (1)]:

(1) $$\text{SSDE}=f_{\text{size}}^{32x}\times {\text{CTDI}}_{\text{vol}}^{32}$$

where $f_{\text{size}}^{32}$ is the conversion factor based on the 32‐cm diameter PMMA phantom for CTDI_vol_ for specific *D_w_* values, determined using [eq. (2)]:

(2) $$f_{\text{size}}^{32x}=4.3781\times e^{-0.0433D_{w}}$$

*D*_w_ was determined for each patient by drawing a freehand region of interest (ROI) around the patient's chest at the central axial slice, while carefully excluding the table. The mean Hounsfield Unit (HU) within the ROI and area of the ROI were recorded and *D_w_* was calculated using equation (3):

(3) $$D_{w}=2\sqrt{\left(1+\frac{{\text{HU}}_{\text{ROI}}}{1000}\right)\frac{{\text{Area}}_{\text{ROI}}}{\pi }}$$

### 2.3 Image quality evaluation

A subset of patients (n = 20) had received consecutive annual screenings between March 2015 and August 2017, with the first examination acquired with FTC and the second examination acquired with TCM. Image quality was evaluated objectively and subjectively for these patients. Noise was measured by recording the standard deviation of the HU values in a 400‐mm^2^ ROI placed in the upper aorta at the level of the carina. Furthermore, contrast to noise ratio (CNR) was measured between the upper aorta and the anterior mediastinal fat located 5‐mm outside of the aorta, using [eq. (4)]:

(4) $$\text{CNR}=\frac{{\text{Mean}}_{\text{upper}\;\text{aorta}}-{\text{Mean}}_{\text{anterior}\;\text{mediastinal}\;\text{fat}}}{\text{Standard}\;{\text{Deviation}}_{\text{anterior}\;\text{mediastinal}\;\text{fat}}}$$

Subjective image quality was evaluated by two board‐certified thoracic radiologists with 15 and 6 yr of experience in reading thoracic CT. They were neither involved in retrieving patient data nor in conducting CT examinations. All 40 studies were de‐identified and displayed on a diagnostic workstation with two monitors using a PACS viewer (Visage Imaging, Inc., Australia) in typical diagnostic radiology reading room lighting conditions. Radiologists were blinded to all scan acquisition techniques. Images were displayed as 1‐mm slices using lung reconstruction kernels and display settings (WL: −550 HU, WW: 1600 HU) for lung evaluation and as 5‐mm slices using soft tissue reconstruction kernels and display settings (WL: 70 HU, WW: 370 HU) for soft tissue evaluation. Radiologists were permitted to adjust window width and window level as necessary to model their typical evaluation of lung screening exams.

The two studies for each patient were displayed side by side, with one monitor displaying the image series acquired with FTC, and the other monitor displaying the image series acquired with TCM. The order of presentation was often switched on the two monitors to prevent pattern recognition of preferred imaging parameters by the radiologist. First, radiologists evaluated the overall diagnostic image quality acceptance for each study using a 3‐point grading scale (Table 2). Next, the radiologists evaluated preference for clinical image quality features specific to lung cancer screening CT including visualization of lung detail, image noise in the lungs, and soft tissue review for incidental findings. Radiologists were asked to provide a verbal response to describe whether they preferred the study on the left monitor or the study on the right monitor, and how strongly they preferred the image. Table 3 describes the verbal response categories they could choose from. The medical physicists conducting the image quality study recorded the verbal response as well as the study accession number displayed on each monitor at the time the question was answered. Using this information, the verbal responses were adapted as scores using a 7‐point grading scale (Table 3).

**Table 2:** Grading scale used in subjective analysis of overall image quality acceptance.

Score	Description
1	Unacceptable, would require a rescan
2	Borderline acceptable, not appropriate for standard protocol
3	Acceptable, can evaluate with confidence

**Table 3:** Grading scale used in subjective analysis of image preference of clinical features.

Blinded radiologist verbal response^a^	Score translated with knowledge of study presentation on monitors^b^	
Response categories	Score	Description
Strongly prefer study on left monitor	−3	Strongly prefer FTC
Slightly prefer study on left monitor	−2	Slightly prefer FTC
Study on left monitor is slightly superior but no preference	−1	FTC is slightly superior but no preference
No visible difference	0	No visible difference
Study on right monitor is slightly superior but no preference	+1	TCM is slightly superior but no preference
Slightly prefer study on right monitor	+2	Slightly prefer TCM
Strongly prefer study on right monitor	+3	Strongly prefer TCM

Abbreviations: FTC, fixed tube current; mA, milliamperes; mAs, milliamperes‐second; TCM, tube current modulation.

^a^ Patient studies acquired with FTC and TCM were presented either on a left or right display monitor, with order of presentation often changed to prevent radiologist recognition of preferred acquisition parameters. Radiologists were not asked to give numerical scores, but rather present their preference verbally using the seven given response categories.

^b^ Medical physicists conducting the observer study recorded which imaging study (FTC or TCM) was presented on which monitor and assigned scores, respectively.

### 2.4 Statistics

A two‐sample t‐test was used to determine whether the patients' age, weight, height, BMI, and *D_w_* showed statistically significant differences between the two patient groups. A two‐sample t‐test was also used to compare CTDI_vol_, SSDE, noise, and CNR between both protocols. Results were considered to be statistically significant for *P* < 0.05. An interobserver agreement for the two radiologists was estimated using the Cohen's Kappa test. A kappa value greater than 0.60 was considered to show substantial interobserver agreement. Furthermore, regression analysis was performed to correlate the relationship between CTDI_vol_, SSDE, noise, CNR, and subjective image quality scores as a function of patient *D_w_* for both FTC and TCM protocols. Pearson correlation coefficients (R^2^) were calculated to evaluate the strength of the relationships, where an R^2^> 0.5 was considered to have a strong relationship.

## 3 Results

Over a period of 29 months, 394 lung cancer screening CT examinations were performed on a Siemens Sensation 16‐slice CT scanner. Of these, 107 were performed with FTC and 287 were performed with TCM following protocol modification. Table 4 lists mean, range, and statistical information for patient demographics and radiation dose metrics. Among the two protocol groups, patient age, weight, height, BMI, and *D_w_* were statistically insignificant (*P* > 0.05), whereas CTDI_vol_, DLP, and SSDE values were statistically significant (*P* < 0.05).

**Table 4:** Patient demographics and radiation dose for examinations performed with FTC and TCM.

		FTC	TCM	
		Mean (range)	Mean (range)	*P* value
Patients	Number (n)	107	287	–
	Age (years)	64.0 (55–77)	64.6 (55–77)	0.39
	Weight (lbs)	176.5 (94.0–266.0)	176.8 (89.0–319.0)	0.95
	Height (inches)	66.7 (57.9–77.0)	66.6 (58.0–78.3)	0.95
	BMI (kg/m^2^)	27.8 (15.8–42.5)	27.95 (13.9–53.1)	0.84
	*D_w_* (cm)	26.7 (18.8–34.9)	26.8 (17.8–37.1)	0.85
Radiation Dose	CTDI_vol_ (mGy)	2.25 (N/A)	2.14 (0.96–4.50)	0.004
	DLP (mGy cm)	95.62 (72.10–143.15)	89.53 (26.93–181.83)	0.01
	SSDE (mGy)	2.70 (2.01–3.72)	2.43 (1.29–4.22)	<0.0001

Abbreviations: BMI, body mass index; CTDI_vol_, volumetric CT dose index; DLP, dose length product; FTC, fixed tube current; SSDE, size‐specific dose estimate; TCM, tube current modulation.

**Fig. 1:**
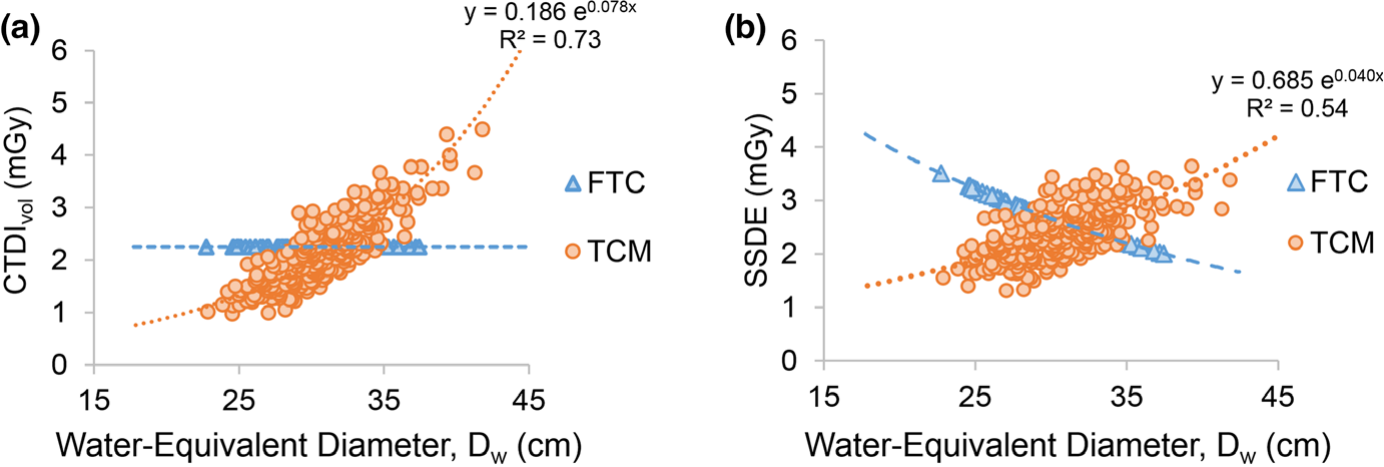
(a) CTDI_vol_ and (b) SSDE as a function of patient *D_w_* for examinations performed with FTC and TCM. CTDI_vol_, volumetric CT dose index; *D_w_*, water‐equivalent diameter; FTC, fixed tube current; TCM, tube current modulation.

All examinations performed with FTC had CTDI_vol_ values equal to 2.25 mGy. Examinations performed with TCM had CTDI_vol_ values ranging from 0.96 to 4.50 mGy (mean 2.14 mGy). Since scans performed with FTC produced the same CTDI_vol_ value for all patients [Fig. 1(a)], SSDE decreased exponentially with *D_w_* (range 2.01–3.72 mGy, R^2^ = 1) [Fig. 1(b)]. Utilizing TCM caused both CTDI_vol_ (range 0.96–4.50 mGy, R^2^ = 0.73) and SSDE (range 1.29–4.22 mGy, R^2^ = 0.54) to increase exponentially with *D_w_* (Fig. 1).

**Fig. 2:**
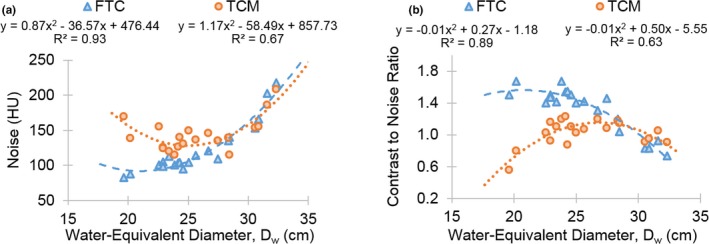
(a) Noise and (b) CNR as a function of patient *D_w_* for examinations performed with FTC and TCM. CNR, contrast to noise ratio; *D_w_*, water‐equivalent diameter; FTC, fixed tube current; TCM, tube current modulation.

**Fig. 3:**
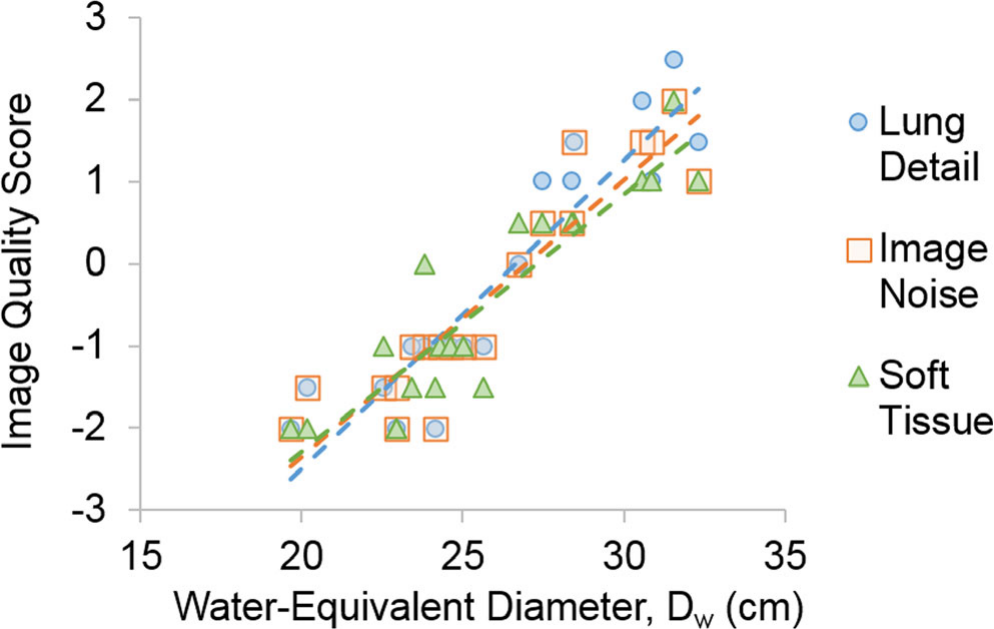
Average subjective image quality scores for evaluation of lung detail, image noise, and soft tissue visualization as a function of patient *D_w_*_._ Scores are described in Table 3. *D_w_*, water‐equivalent diameter.

Noise and CNR were dependent on patient size and acquisition method (*P* < 0.005) and demonstrated two‐order polynomial relationships with *D_w_* (R^2^> 0.50) (Fig. 2). With the FTC protocol, noise increased and CNR decreased with *D_w_*. With the TCM protocol, noise decreased, then increased, and CNR increased, then decreased, with *D_w_*, demonstrating noise and CNR measurements were only superior with the TCM protocol for larger sized patients with *D_w_*> 28.4 cm. The calculation of the kappa coefficient showed strong interobserver agreement (k = 0.64) between the radiologist blinded to the protocol used. Image quality scores for clinical features averaged over both radiologists increased with *D_w_* for visualization of lung detail (R^2^ = 0.85), overall image noise (R^2^ = 0.85), and soft tissue review for incidental findings (R^2^ = 0.82) (Fig. 3). Considering a score of −3 represents Strongly prefer FTC, and a score of + 3 represents Strongly prefer TCM, the increasing trend in Fig. 3 demonstrates that radiologists preferred images acquired with FTC (score < 0) for smaller sized patients with *D_w_* < 28.4 cm and with TCM (score> 0) for larger sized patients with *D_w_*> 28.4 cm. However, both radiologists scored the overall image quality acceptance for all images, acquired with FTC and TCM, as 3: Acceptable, can evaluate with confidence.

## 4 Discussion

To our knowledge, no studies have reported CTDI_vol_ and SSDE for a variety of patient sizes examined for lung cancer screening with LDCT on a Siemens Sensation 16‐slice scanner. With TCM, CTDI_vol_ ranged from 0.96 to 4.5 mGy, which is within the approximate expected CTDI_vol_ ranges for all CT scanners of 0.25–5.6 mGy reported by the AAPM. It is important to note that the CMS specifies the CTDI_vol_ should be below 3.0 mGy for an average sized patient. In our findings, patients who received a CTDI_vol_ above 3.0 mGy had a *D_w_* greater than 33.0 cm. All patients with a *D_w_* greater than 33.0 had a BMI greater than 36.9 kg/m^2^, considered to be obese.

The subjective image quality assessment performed with two experienced thoracic radiologists confirmed the recommended scan protocol for the Siemens Sensation 16‐slice scanner produced acceptable clinical image quality for the evaluation of lung cancer screening for high‐risk patients ranging from underweight to obese body sizes. It is interesting to note this older 16‐slice scanner without iterative reconstruction was able to produce acceptable radiation dose values and image quality evaluation for a variety of patients examined with a low‐dose screening protocol, and therefore, hospitals should not assume a dose reduction tool such as iterative reconstruction is necessary to achieve an effective low‐dose lung cancer screening CT program.

When FTC techniques were utilized, the CTDI_vol_ and thus radiation output from the scanner were the same for all patients. As a result, smaller patients, having less mass than larger patients, received greater radiation absorbed dose, demonstrated by SSDE decreasing exponentially as the patient size increased [Fig. 1(b)]. Among patients scanned with the FTC protocol, the smallest sized patient (weight, 94.0 lbs) had an SSDE of 3.5 mGy, while the largest sized patient (weight, 244.0 lbs) had an SSDE of 2.0 mGy. The greater SSDE is unnecessary for smaller sized patients who do not require increased photon transmission as larger sized patients do. This approach of delivering the same radiation output to all patients independent of their size reflects back to when scan techniques were not modified for small vs large patients and has been widely replaced by recommendations for TCM.^13^, ^14^, ^15^, ^16^, ^17^

When the TCM protocol was utilized, smaller patients received less SSDE than larger patients, demonstrated by SSDE increasing exponentially as patient size increased [Fig 1(b)]. Among patients scanned with TCM, the smallest sized patient (weight, 97.0 lbs) had an SSDE of 1.6 mGy, and the largest sized patient (weight 266.0 lbs) had an SSDE of 3.4 mGy. Another study assessing SSDE in CT of the torso reported patient size had an effect on CTDI_vol_ but not on SSDE, concluding that increasing the scanner output for larger patients would not necessarily increase the mean absorbed dose to the patients.^26^ Alternatively, our study focusing on the relatively less attenuating chest region observed a statistically significant exponential increase in SSDE as a function of patient size, indicating larger sized patients experienced greater mean absorbed dose than smaller sized patients. These results are also supported by a previous study on patient size and impact of attenuation‐based AEC on low‐dose lung cancer screening protocols.^27^ Our finding that SSDE increases for larger sized patients is critical for other studies estimating long‐term stochastic risks in the lung cancer screening population, which requires a multi‐vendor investigation of different AEC systems.

**Fig. 4:**
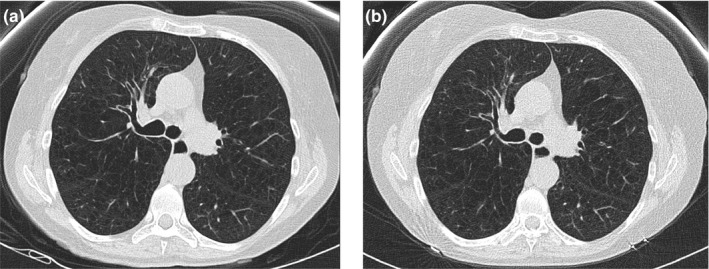
Axial images of the smallest sized patient who received annual lung cancer screening CT examinations scanned with (a) FTC the first year and (b) TCM the second year. FTC, fixed tube current; TCM, tube current modulation.

**Fig. 5:**
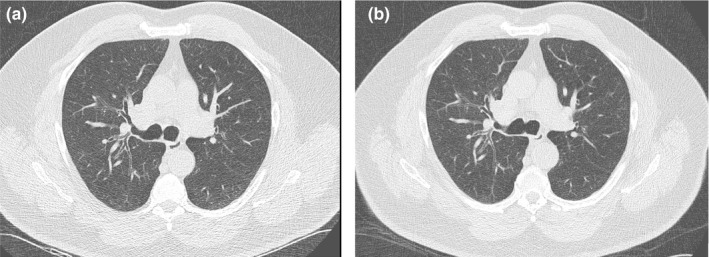
Axial lung images of the largest sized patient who received annual lung cancer screening CT examinations scanned with (a) FTC the first year and (b) TCM the second year. FTC, fixed tube current; TCM, tube current modulation.

Relative to the FTC protocol, objective and subjective image quality decreased for smaller sized patients with the use of TCM. In the smallest patient who received consecutive exams with FTC and TCM, both radiologists preferred images acquired with FTC [Fig. 4(a)] rather than with TCM [Fig. 4(b)]. Figure 4 demonstrates less image noise, better low contrast resolution, and reduced artifacts in the FTC image (a) compared to the TCM image (b). CNR was also superior for the FTC images (CNR = 1.67) compared to the TCM images (CNR = 1.21). This was expected, as the study acquired with FTC utilized a lower tube voltage (100 kVp) and a higher CTDI_vol_ (2.25 mGy) than the same patient's study acquired with TCM (100 kVp, 1.21 mGy). However, despite the inferior image quality metrics, all TCM images were still deemed clinically acceptable. Both objective and subjective image quality measurements improved for larger sized patients with the use of TCM. With TCM, CTDI_vol_ values increased for larger sized patients, reducing image noise with an acceptable compromise in increased radiation dose. This occurred with a simultaneous increase in tube voltage from 100 kVp to 120 kVp, which reduces image contrast. For the largest sized patient who received consecutive exams with fixed and modulating tube current, both radiologists preferred image quality of the images acquired with TCM [Fig. 5(b)] rather than with FTC [Fig. 5(a)]. It is evident in Fig. 5 that the TCM image (b) has significantly less streak artifacts than the FTC image (a). CNR was also superior for the TCM images (CNR, 1.13) compared to the FTC image (CNR, 0.93). For this patient, the study acquired with TCM utilized a higher CTDI_vol_ (3.2 mGy) than the study acquired with FTC (2.25 mGy). However, the increase in radiation exposure is warranted given the large patient size and recommendations to increase techniques for larger sized patients.^8^, ^11^, ^12^

Based on the SSDE and image quality results in this study, utilizing tube current modulation for all patients is ideal, as both CTDI_vol_ and SSDE were reduced for small patients and increased for large patients, and image quality was acceptable for all TCM examinations. This opposes recommendations from some CT scanner manufacturers who currently recommend performing low‐dose lung cancer screening CT with fixed tube current to ensure low doses for all patients.^11^ Furthermore, it is possible that CT technologists and CT applications specialists may assume a fixed dose protocol is a simple solution to a low‐dose screening program examining patients of all sizes, as we initially observed in our hospital.

When using attenuation‐based TCM, it is important to consider situations in which high attenuating materials, such as metal implants, would drive the tube current higher, and so it is beneficial to set a maximum tube current setting. Furthermore, setting a maximum tube current value can also ensure appropriate low‐dose exams by limiting the CTDI_vol_ in the case of a very large patient.

Our study has limitations. The initial FTC examinations were acquired with a tube voltage of 100 kVp rather than 120 kVp. Since a lower tube voltage offers improvement in image contrast, the comparison of CNR and subjective image quality is not only due to changes in x‐ray output but also increased photon absorption at a lower kVp. However, it is likely that the reduced tube voltage of 100 kVp would offer an improvement in low contrast soft tissue assessment but not as much in high contrast lung detail.^28^ Furthermore, if performing an assessment with equivalent tube voltages, we would expect for FTC exams acquired with 120 kVp to produce inferior CNR measurements compared to those measured in our study with 100 kVp, and therefore, the differences in image quality observed in this study would have been even greater. In addition, increasing the tube voltage from 100 kVp to 120 kVp reduces the beam hardening effect and artifacts such as streaking, which would be expected to be greater in the upper thorax and in larger sized patients. However, artifact evaluation was not included in the image quality assessment.

We also did not investigate lung cancer screening protocols using other vendors. However, all scan protocols should benefit from the use of TCM as it modifies the tube current output accordingly for smaller or larger patients and removes the need for manual technique adjustment by the technologist when scanning small‐ vs large‐sized patients. Furthermore, although patient weight is often readily available, it cannot be directly correlated to water‐equivalent diameter, as there are variable human somatotypes. This has been shown to be especially true of the thorax where there is higher variation along the z‐axis.^25^ There remains a need to assess other vendor AEC systems in order to describe the effect of patient size on SSDE for future risk estimates.

## 5 Conclusion

In summary, SSDE takes into account the size of the patient and provides a more accurate reflection of patient dose than CTDI_vol_. When considering the SSDE for patients of different body sizes, a protocol that balances diagnostic acceptability with dose reduction should be performed. We confirmed there was an unnecessary increase in SSDE for small‐sized patients and reduction in SSDE for large‐sized patients when scanning with FTC techniques. Scanning with TCM produced more favorable dose output based on patient size and is supported by current AAPM recommendations.

Using TCM to adjust scanner output for lung cancer screening with LDCT resulted in an exponential relationship between patient size, CTDI_vol_, and SSDE, indicating that increasing scanner output for larger patients also increased the mean absorbed dose to these patients. Furthermore, examinations performed with TCM received superior image quality measurements for larger sized patients (*D_w_* > 28.4 cm).

In consideration of enforcing the ALARA (As Low As Reasonably Achievable) principle, all patients examined with lung cancer screening CT should be scanned with TCM for optimal clinical practice, considering its ability to automatically modify radiation output as necessary and yield acceptable image quality by interpreting radiologists.

## IRB statement

This work was conducted with permission granted by our Institutional Review Board (IRB) under study number IRB201500809 titled “Low‐dose Computed Tomography Screening for Lung Cancer: Initial screening experience at an academic medical center.” Full waiver of informed consent was approved, and therefore, subjects were not informed nor consented for retrospective examination data collection.

## Conflicts of interest

The authors have no conflicts of interest.
